# Predicting the Specificity- Determining Positions of Receptor Tyrosine Kinase Axl

**DOI:** 10.3389/fmolb.2021.658906

**Published:** 2021-06-14

**Authors:** Tülay Karakulak, Ahmet Sureyya Rifaioglu, João P. G. L. M. Rodrigues, Ezgi Karaca

**Affiliations:** ^1^Izmir Biomedicine and Genome Center, Izmir, Turkey; ^2^Izmir International Biomedicine and Genome Institute, Dokuz Eylul University, Izmir, Turkey; ^3^Institute of Molecular Life Sciences, University of Zurich, Zurich, Switzerland; ^4^Department of Pathology and Molecular Pathology, University Hospital Zurich, Zurich, Switzerland; ^5^Swiss Institute of Bioinformatics, Lausanne, Switzerland; ^6^Department of Electrical – Electronics Engineering, İskenderun Technical University, Hatay, Turkey; ^7^Department of Structural Biology, Stanford University School of Medicine, Stanford, CA, United States

**Keywords:** protein selectivity, sequence analysis, molecular dynamics, Axl, HADDOCK

## Abstract

Owing to its clinical significance, modulation of functionally relevant amino acids in protein-protein complexes has attracted a great deal of attention. To this end, many approaches have been proposed to predict the partner-selecting amino acid positions in evolutionarily close complexes. These approaches can be grouped into sequence-based machine learning and structure-based energy-driven methods. In this work, we assessed these methods’ ability to map the specificity-determining positions of Axl, a receptor tyrosine kinase involved in cancer progression and immune system diseases. For sequence-based predictions, we used SDPpred, Multi-RELIEF, and Sequence Harmony. For structure-based predictions, we utilized HADDOCK refinement and molecular dynamics simulations. As a result, we observed that (i) sequence-based methods overpredict partner-selecting residues of Axl and that (ii) combining Multi-RELIEF with HADDOCK-based predictions provides the key Axl residues, covered by the extensive molecular dynamics simulations. Expanding on these results, we propose that a sequence-structure-based approach is necessary to determine specificity-determining positions of Axl, which can guide the development of therapeutic molecules to combat Axl misregulation.

## Introduction

The functional identification of proteins is essential to understand the grounds of innate cellular processes. Several computational tools have been deployed to annotate protein function from ever-accumulating protein sequences (Friedberg, 2006). These approaches aim to define functionally important residues through comparative sequence analysis (Whisstock and Lesk, 2004). Resolving the functionally key amino acids is particularly interesting, as modulation of these residues holds a great potential to design protein-based therapeutics (Moll et al., 2016). Such key amino acids can be identified upon searching for conserved positions across different species. Alternatively, within a species, one could look for the differentially mutated amino acid positions of closely-related protein families, i.e., paralogs (Gogarten and Olendzenski, 1999; Mirny and Gelfand, 2002; Chagoyen et al., 2016). In paralogs, some mutations are evolved to act as specificity-determining positions (SDPs) for regulating selective protein interactions (Rausell et al., 2010; Sloutsky and Naegle, 2016). Thus, SDPs are often ascribed to the specialized functions of proteins (Capra and Singh, 2008; Chakraborty and Chakrabarti, 2015; Wong et al., 2015). SDPs can either select a binding partner (partner-selecting) or tune the affinity of a protein toward different ligands (affinity-tuning) (Chagoyen et al., 2016; Sloutsky and Naegle, 2016; Pitarch et al., 2020).

During the last three decades, several sequence-based SDP predictors have been proposed (Pirovano et al., 2006; Chakrabarti and Panchenko, 2008; Chakraborty and Chakrabarti, 2015; Chagoyen et al., 2016). These methods rely on the application of different machine learning techniques, which can be grouped into entropy-, evolution-, and feature-based (Teppa et al., 2012). The majority of these methods expand on the use of a precalculated multiple sequence alignment (MSA) file. The entropy-based methods compute the variability of specific amino acid positions in an alignment of related protein sequences, allowing the identification of highly varying positions (Kalinina et al., 2004; Ye et al., 2006; Feenstra et al., 2007). As an example, SDPpred uses mutual information entropy scores to predict SDPs (Kalinina et al., 2004). The evolutionary-based methods, on the other hand, use substitution matrices or phylogenetic trees to calculate residue-based variability scores (del Sol Mesa et al., 2003; Pazos et al., 2006; Capra and Singh, 2008). The evolutionary-based method Xdet, for example, combines the substitution matrix with GO or EC annotations, together with the available interactome data (Pazos et al., 2006). Different than the other sequence-based methods, Xdet can provide partner-specific SDPs, though, it only works on large protein families (Pitarch et al., 2020). Finally, the feature-based methods perform feature extraction of each amino acid position. The extracted feature vectors are fed into a classifier, such as random forest, support vector machine or neural network (Ahmad and Sarai, 2005; Wong et al., 2015). For instance, Ahmad and Sarai proposed a position-specific scoring matrix-based SDP prediction of DNA binding proteins (Ahmad and Sarai, 2005). Here, each residue is represented as a feature vector by using its and its neighbors’ conservation scores. Then, the feature vectors are processed by a neural network classifier to categorize the input residues as SDP or non-SDP for DNA binding. As the sequence-based SDP prediction methods do not use heavy input data, they are computationally efficient. However, the application of these methods is rather limited as they are mostly trained with small sequence datasets with classical machine learning algorithms.

The available structure-based SDP prediction methods make use of the core-support-rim model, as proposed by Levy. According to this model, the protein-protein interaction surface can be dissected into three, as: (i) the core; the amino acids, which get buried upon complexation, (ii) the support; the residues, which are buried in the uncomplexed state and become more buried upon complexation, (iii) the rim; the amino acids, which stay solvent accessible both in free and complexed states (Levy, 2010). In a recent work of Ivanov et al., this definition was used to discriminate SDPs of four paralog protein families (Ivanov et al., 2017). Here, the authors structurally modeled and analyzed all paralog interactions, for which the experimental affinities were at hand. Their analysis showed that SDPs are located at the rim, where they form strong electrostatic (charge-charge) interactions (Chakrabarti and Janin, 2002; Ivanov et al., 2017). Other groups utilized atomistic molecular dynamics simulations to trace partner-selecting paralog interactions. For example, van Wijk et al. demonstrated that a single salt bridge is the key determinant for selective ubiquitin-conjugating enzyme (E2) and ubiquitin ligase (E3) interactions (van Wijk et al., 2012). Being at the rim of E2-E3 surface, the partner-selecting role of this salt bridge was validated by mutagenesis and yeast two-hybrid screening. Another recent example explored how protocadherins specifically find their partners to polymerize, which is an essential mechanism for neuronal development. For this, Nicoludis et al. combined molecular dynamics simulations with evolutionary coupling information (Nicoludis et al., 2019). Compared to the sequence-based SDP prediction methods, the structure-based approaches provide a refined and thus an experimentally testable SDP set. However, these approaches generally require expertise in computational structural biology tools and depending on the size of the system, they could be computationally intensive.

As the sequence- and structure-based methods have different advantages, we chose a model system to map the prediction landscape of these approaches. For this, we concentrated on a paralogous protein receptor tyrosine kinase family (TAM), made by Tyro3, Axl, and Mer proteins. TAM receptors, like the other receptor tyrosine kinases, are activated through their interactions with extracellular proteins, triggering receptor dimerization and autophosphorylation of their kinase domains (Rothlin and Lemke, 2010). Earlier studies identified two related proteins, the growth arrest-specific protein 6 (Gas6) and vitamin K-dependent protein S (Pros1) as TAM ligands (Hafizi and Dahlbäck, 2006). The binding of these ligands to TAM leads to downstream activation of diverse signaling pathways (Wium and Paccez, 2018). Besides Gas6/Pros1, three other ligands (tubby, tubby-like protein and galactin-3) were shown to bind to TAM proteins (Myers et al., 2019). These structures are neither sequence- nor structure-wise related to Gas6 and Pros1. This suggests that they bind to TAM family by using a different mechanism compared to Gas6 and Pros1. As there is little information on the binding profiles of these new ligands, in this work, we focused only on TAM:Gas6/Pros1 interactions.

TAM receptors share 52–57%, while Gas6/Pros1 share 40% pairwise sequence similarity. Across TAM members, Pros1 binds to Tyro3 and Mer, while it cannot bind to Axl. Gas6 binds to all three receptors with the highest affinity toward Axl (Hafizi and Dahlbäck, 2006; Yanagihashi et al., 2017). Among the different combinations, the Axl:Gas6 interaction is particularly interesting given its involvement in numerous types of signaling pathways (e.g., tumor-cell growth, metastasis, epithelial to mesenchymal transition, drug resistance, etc.) (Zhu et al., 2019). Relatedly, Axl aberrant regulation was shown to lead to different types of cancer and infectious diseases (Van Der Meer et al., 2014), as well as to promote SARS-CoV-2 entry into cell (Wu et al., 2017; Wium and Paccez, 2018; Wang et al., 2021). Although the structure Axl:Gas6 complex is resolved, Axl’s ligand-selecting residues is still unknown. To help to close this knowledge gap, we used three sequence-based SDP predictors, SDPpred, Multi-RELIEF (both feature-based), and Sequence Harmony (entropy-based) to map Axl partner-selecting SDPs. In addition, we analyzed the selective Axl:ligand interactions, by using simple refinement and extensive molecular dynamics simulations.

## Results

### Axl:Gas6 Interface

**FIGURE 1:**
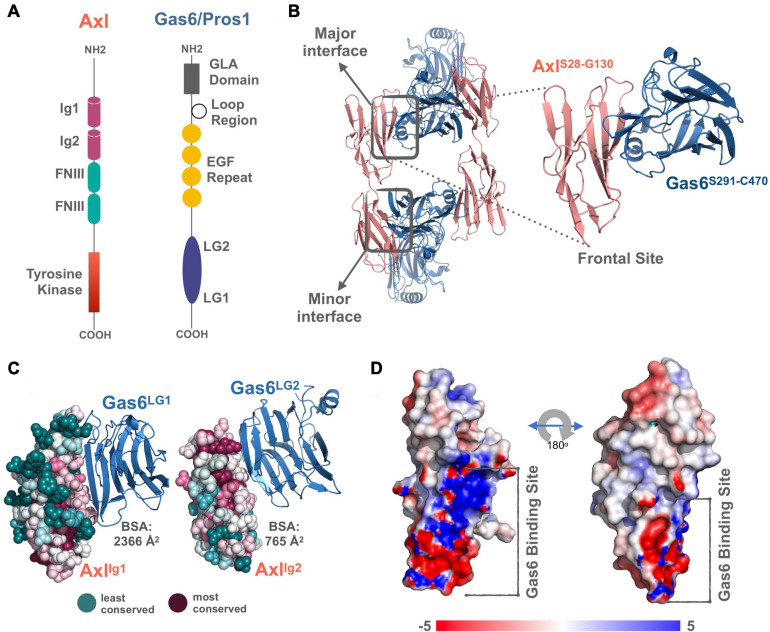
**(A)** The domain organization of TAM family and its ligands, Gas6 and Pros1. TAM family consists of Ig1, Ig2, two FNIII, and tyrosine kinase domains (Linger et al., 2008; Lemke, 2013). Gas6 and Pros1 are composed of GLA domain, loop region, EGF Repeat, and LG2, LG1 domains (Linger et al., 2008; Lemke, 2013). **(B)** Axl(Ig1-Ig2):Gas6(LG1-LG2) interaction involves two interfaces: The major interface is formed between Axl-Ig1:Gas6-LG1 and the minor one is established among Axl-Ig2:Gas6-LG1 [PDB ID: 2C5D, Sasaki et al. (2006)]. The inset represents the charged frontal side of the major interface (Axl is depicted in pink cartoon, whereas Gas6 is represented in purple cartoon). **(C)** Conservation scores of Axl residues predicted via ConSurf webserver (Glaser et al., 2003; Landau et al., 2005; Ashkenazy et al., 2016). The most conserved sites are colored with deep purple and the least conserved ones with deep teal. **(D)** Electrostatic potential of Axl:Gas6 interacting site. The color scale ranges from -5 (red) to 5 (blue). One side of Axl’s Gas6 binding surface is heavily charged, while the other side is composed of neutral amino acids.

TAM receptors share two immunoglobulin (Ig)-like, two fibronectin type III domains (FNIII), followed by a single-pass transmembrane helix, and an intracellular kinase domain (Figure 1A). TAM ligands, Gas6 and Pros1 contain an N-terminal gamma-carboxyglutamic acid (GLA) domain, four epidermal growth factor-like (EGF) repeats, and two laminin G (LG)-like domains (Figure 1A). The crystal structure of Axl:Gas6 interaction is the only available TAM:ligand structure [PDB ID: 2C5D (Sasaki et al., 2006)]. In the Axl:Gas6 structure, two Ig-like domains of Axl interact with two LG-like domains of Gas6, without involving any receptor-receptor or ligand-ligand interactions (Figure 1B). Axl and Gas6 interact through two symmetric copies of major and minor interfaces, burying 2366 Å^2^ and 765 Å^2^ surface areas, respectively (Figures 1B,C). While the minor interface is highly conserved across TAM, the major interface is not. The major interface is spatially segregated into a frontal site, involving a series of charged residues, and a hydrophobic distal site (Figure 1D; Sasaki et al., 2006). The segregated characteristics of the major interface contribute to its ligand selection, as well as to Axl’s high affinity toward Gas6 (Sasaki et al., 2006). Thus, for studying the ligand selectivity of Axl, we focused on the major Axl:Gas6 interface (Figure 1B-inset).

### Sequence-Based Axl SDP Predictions Agree in One Residue

Among the available sequence-based SDP predictors, we selected three methods to probe Axl ligand selectivity (Supplementary Table 1). These algorithms, i.e., SDPpred, Sequence Harmony, and Multi-RELIEF, were selected based on their widespread use and their availability as a web service (Kalinina et al., 2004; Feenstra et al., 2007; Ye et al., 2008). Initially, to analyze the TAM sequences, the mammalian (human, mouse, rat, pig, chimpanzee) TAM Ig1 sequences were retrieved from UniProtKB (The UniProt Consortium, 2018). These sequences were grouped into Axl and Tyro3 & Mer sequence groups. The MSA of each group was constructed with Clustal Omega (Sievers and Higgins, 2017). For each approach, MSAs were formatted according to the requirements of the webservers. As earlier studies showed that partner-selecting SDPs are located at the rim of protein-protein interfaces, we filtered out the sequence-based SDP predictions by keeping the positions corresponding to the rim of the Axl:Gas6 complex (Ivanov et al., 2017). Within this framework, SDPpred predicted 19 SDPs, five of which (T46, R48, Q50, D84, K96) were at the rim of Axl:Gas6. The majority of the SDPpred predictions corresponded to the non-interacting regions of the Axl:Gas6 complex (as calculated by the EPPIC web server, Duarte et al., 2012). The same trend was observed for Multi-RELIEF, which contained two rim Axl amino acids out of 15 SDP predictions (R48, E70). In the case of Sequence Harmony, the minority of the predictions (4/15) were located at the rim of Axl:Gas6 (T46, R48, Q50, K96). As such, the combined Axl SDP list, predicted by these three webservers became T46, R48, Q50, E70, D84, K96, where they only agreed on R48. The complete list of the SDP predictions is provided under Supplementary Table 2.

### Axl Selectivity Is Regulated by Salt Bridges

**FIGURE 2:**
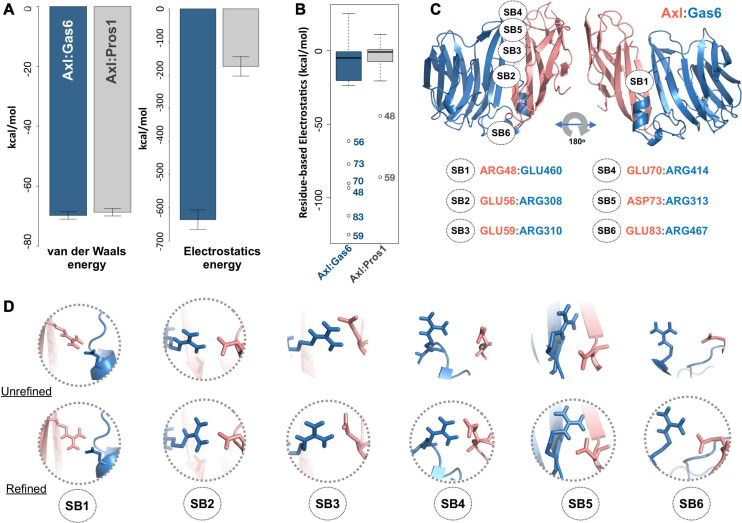
**(A)** The mean van der Waals and electrostatics energetics of the top scoring Axl:Gas6 and Axl:Pros1 major interfaces calculated by HADDOCK. The mean van der Waals energies of Axl:Gas6 (blue) and Axl:Pros1 (gray) are –69.8 ± 1.2 kcal/mol and –68.9 ± 2.2 kcal/mol, respectively. The mean electrostatics of Axl:Gas6 and Axl:Pros1 are –635.8 ± 29.3 kcal/mol and –173.5 ± 21.4 kcal/mol, respectively. **(B)** Residue-based electrostatics contribution of Gas6-facing Axl residues. Axl residues behaving differently than the rest of the population are R48, E59, E70, D73, E83 in the case of Axl:Gas6 (blue), and E48, E59 in the case of Axl:Pros1 (gray). The whiskers were computed with a whisker length of 2xIQR. **(C)** The distribution of the salt bridges (SBs) across Axl:Gas6 interface. Axl is represented in light pink and Gas6 in marine blue. Only SB1 is located on the back and rather neutral side of the complex. This and all the structural images were generated with PyMOL molecular visualization software (Schrödinger, 2015). **(D)** The arrangement of the potential SB-making residues in the case of crystal (first row, pdb id: 2C5D) and HADDOCK-refined (second row) Axl:Gas6 complex. The pairs forming SBs are encircled.

To study partner-selecting Axl SDPs, we modeled the three-dimensional structure of Axl:Pros1 (Ig1:LG1) complex, to use it as the negative (non-binder) control. We refined the two Axl:ligand complexes with HADDOCK 2.2 webserver (van Zundert et al., 2016). We chose HADDOCK, since it provides a user friendly web service to carry out the analysis proposed here. When used for refinement, HADDOCK skips docking stages and performs several independent short molecular dynamics simulations in explicit solvent. The top-scoring Axl complexes, ranked by the HADDOCK score, differed mostly in interface electrostatics: Axl:Gas6 has ∼3.6 times better electrostatics energy than Axl:Pros1 (−635.8 ± 29 kcal/mol vs. −173.5 ± 21 kcal/mol) (Figure 2A). Other interface features and energy terms, such as buried surface area and van der Waals energies, were comparable between the complexes. These results underscore that Axl selectivity is mainly driven by the electrostatics interactions. We analyzed per-residue electrostatics of interfacial Axl residues (31 for the Axl:Gas6 complex and 27 for Axl:Pros1) (Figure 2B). In the case of Axl:Gas6, Axl R48, E56, E59, E70, E73, E83 contributed to the interface electrostatics the most (Figure 2B). Being at the rim of the Axl:Gas6 complex, these residues formed six different salt bridges (Figure 2C and Supplementary Table 2). As introduced earlier, previous studies have shown that the ligand-selecting SDPs are rim amino acids, capable of forming opposing charge interactions. This made these salt bridge forming residues the perfect SDP candidates. Among these six salt bridges (SBs), SB2-6 were located on the charged frontal side of the complex (Figure 2C, left). Interestingly, SB1 and SB3 (mediated by R48, E59) were also present at the Axl:Pros1 interface. We, therefore, eliminated R48, E59 from the initial Axl SDP list. This left E56, E70, D73, E83 Axl residues as the strongest partner-selecting SDPs. Here, we should note that SB3-SB6 were not present in the Axl:Gas6 crystal structure (Figure 2D). The proper establishment of these salt bridges was secured only after the HADDOCK refinement. Finally, if E56, E70, D73, E83 were Gas6-selective, their positions should be substituted with different amino acids in Tyro3 and Mer. This turned out to be the case for E70 and E83, leaving those as the final HADDOCK-based Axl SDP predictions (Table 1).

**TABLE 1:** Positional sequence comparison of R48, E56, E70, D73, E83. E56 and D73 are conserved in both Axl and Tyro3 (shown in bold).

Axl	Tyro3	Mer
R48	N63	N114
**E56**	**E70**	Q124
E70	Q85	L138
**D73**	**D87**	H141
E83	-	D151

**FIGURE 3:**
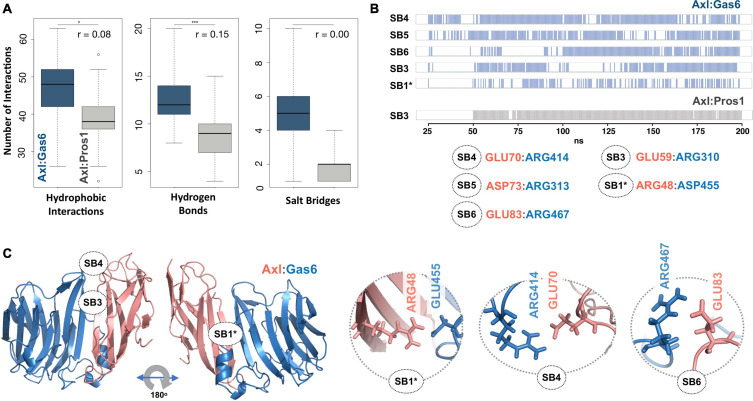
**(A)** The distributions of hydrophobic contact (left), hydrogen bond (middle) and SB numbers (right) for Axl:Gas6 (marine blue) and Axl:Pros1 (light gray) simulations. The distributions are interpreted with box-and-whisker statistics. The associations between two Axl:Gas6 and Axl:Pros1 distributions were reflected in r values, according to which the SB distributions do not have anything in common. **(B)** Consistently and stably observed SBs formed in Axl:Gas6 and Axl:Pros1 simulations. Each row indicates the observation frequency of the indicated SB. The frequency data came from replica 1 simulations (Table 2). The SB numbering follows the ones used in Figure 2C. SB1 is denoted with * as in this case R48 couples with a different Gas6 residue than observed in Figure 2C. **(C)** The positioning of potential SDPs on Axl:Gas6 structure predicted by molecular dynamics analysis.

To explore the time-dependent interaction profiles of Axl:Gas6 and Axl:Pros1, we carried out molecular dynamics (MD) simulations of the HADDOCK-refined Axl complexes. Even though running MD simulations requires expertise, we used it to gain the highest resolution information on our system. For each complex, we ran four independent (replica) MD simulations, totaling 1.6 microseconds. The analysis of these trajectories showed that the Axl:Gas6 complex is more stable than Axl:Pros1, as reflected in the lower root mean square deviation (RMSD) (0.15 ± 0.01 nm vs. 0.23 ± 0.03 nm) (Supplementary Figure 1), and radius of gyration profiles (Supplementary Figure 2). To perform a more in-depth analysis of the interactions between Axl and its ligands, we calculated the inter-molecular hydrophobic, hydrogen bonds and salt bridges formed during the simulations by using the *interfacea* python package (Figure 3A). When we pooled the interaction data of each Axl complex, we observed that Axl:Pros1 contained a fewer number of contacts in all interaction types. The most significant difference between Axl:Gas6 and Axl:Pros1 interaction distributions was observed in the case of salt bridges. Axl:Gas6 trajectories reflected, on average, four to five stable salt bridges, where this number dropped to two in the case of Axl:Pros1 (Figure 3A, right panel). We then looked for the salt bridges, which were seen in four different trajectories consistently for more than 25% of the simulation time (Table 2). Here, our assumption was that the SDP positions should form stable salt bridges within a trajectory and should be observed consistently across four trajectories. These criteria left us with five salt bridges, four of which were the same as the ones selected by the HADDOCK refinement: E70:R414^*Gas6*^ (SB4), D73:R313^*Gas6*^ (SB5), E83:R467^*Gas6*^ (SB6), E59:R310^*Gas6*^ (SB3) (listed in the decreasing observation frequency in Figure 2B). E56-mediated SB2, coming from our HADDOCK refinement analysis was observed only in one replica, indicating that it could be coincidental (Table 2). As another surprising outcome, R48 of SB1 formed a stable and consistent salt bridge with D455^*Gas6*^, instead of E460^*Gas6*^, which was suggested by the HADDOCK refinement. Interestingly, E460^*Gas6*^ has also a glutamic acid correspondence on Pros1, while in D455^*Gas6*^ matches with an alanine in Pros1. In the case of Axl:Pros1, only E59:K314^*Pros1*^ was observed in a statistically significant manner, which corresponds to SB3 of Axl:Gas6 (Figure 3B and Table 2). These observations left us with four possible selective salt bridges, three of which were formed by the positions unique to Axl: R48, E70, E83 (Table 1). Our across-ortholog comparison revealed that R48, E70, E83 are all conserved, supporting the SDP candidacy of these positions (Supplementary Figure 3). The spatial distribution of the final list of salt bridges formed by these residues is illustrated in Figure 3C.

**TABLE 2:** SBs formed in parallel **(A)** Axl:Gas6 and **(B)** Axl:Pros1 simulations.

(A)	Axl Resi	Gas6 Resi	Axl:Gas6-replica #1 (%)	Axl:Gas6-replica #2 (%)	Axl:Gas6-replica #3 (%)	Axl:Gas6-replica #4 (%)
**SB4**	**70**	**414**	**81.14**	**34.57**	**55.41**	**70.57**
**SB5**	**73**	**313**	**72.86**	**81.71**	**69.43**	**76.86**
**SB6**	**83**	**467**	**60.29**	**48.00**	**68.57**	**71.14**
**SB3**	**59**	**310**	**59.43**	**32.86**	**83.14**	**56.86**
SB1*	**48**	**455**	**43.71**	**44.00**	**53.43**	**30.57**
SB2	56	308	–	–	–	25.14

**(B)**	**Axl**	**Pros1**	**Axl-Pros1-replica #1 (%)**	**Axl-Pros1-replica #2 (%)**	**Axl-Pros1-replica #3 (%)**	**Axl-Pros1-replica #4 (%)**

**SB3**	**59**	**314**	**97.67**	**97.67**	**94.33**	**94.33**
SB2	59	316	–	–	–	66.66
SB1	48	465	64.67	51.67	–	33.00

Each row indicates the observation frequency of the denoted salt bridge. The SB numbering follows the ones used in Figure 2C. The consistent and stable SBs are marked in bold. * corresponds to the SB1* presented in Figure 3B.

## Discussion

In this work, we used three sequence-based SDP predictors, namely, SDPpred, Multi-RELIEF, and Sequence Harmony to map Axl’s ligand-selecting SDPs. Next to these approaches, we also carried simple refinement and extensive MD simulations of Axl:ligand interactions. As the primary outcome of this exercise, we found that the sequence-based SDP predictors largely overpredict the potential SDP positions. Hence, we used available literature data to filter out the structurally non-viable ligand-selecting SDPs. As a result, the three methodologies in combination proposed six SDPs, where they agreed only on R48. Our HADDOCK-refinement-based approach suggested R48 as a strong electrostatic contributor to the Axl:Gas6 interface. Though, by only following HADDOCK refined structures, we had to eliminate R48 from the potential SDP list, as it significantly contributed to the Axl:Pros1 interaction energetics too. Elaborate MD simulations were necessary to rescue R48’s SDP candidacy. During our MD simulations, R48 formed a new salt bridge, which was neither observed in the crystal nor in the HADDOCK refined complexes. In the end, HADDOCK refinement proposed four selective SBs, three of which were supported by the MD simulations. Checking the evolutionary variance of MD-deduced SDP positions suggested R48, E70, and E83 as the strongest Axl SDP candidates. Strikingly, Multi-RELIEF (plus the rim information) could predict two of these (R48, E70) without running extensive simulations.

**FIGURE 4:**
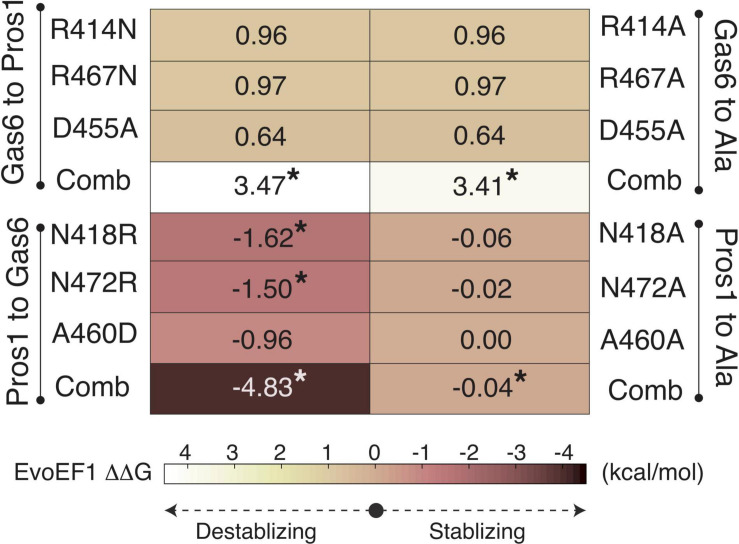
EvoEF1 △△G predictions for the selective Gas6-to-Pros1 or Pros1-to-Gas6 mutations. R414, R467, and D455 correspond to SB4, SB6, and SB1* forming residues, as presented in Figure 3B. The significant changes are marked with *. The stabilizing and destabilizing mutation color scheme ranges from –4 (brown) to 4 (yellow). Gas6-to-Ala and Pros1-to-Ala mutations were run as a control. Comb refers to combined mutations.

To validate R48, E70, and E83 as the ligand-selecting Axl SDPs, we artificially mutated the SB1, SB4, and SB6 forming Gas6 residues their Pros1 counterparts, and vice versa, by using EvoEF1 (Pearce et al., 2019; Figures 3, 4). EvoEF1 is a machine learning approach, poised to calculate the impact of point mutations across protein-protein interfaces. According to EvoEF1, Axl:Gas6 interaction stability was significantly reduced when individual and combined Gas6-to-Pros1 and Gas6-to-alanine mutations were imposed. On the other hand, individual and combined Pros1-to-Gas6 mutations led to a significant increase in the stability of Axl:Pros1 complex (Figure 4). These findings underscore the vitality of SB1, SB4, and SB6 to the formation of Axl:Gas6 complex (Figure 3C).

### Future of the SDP Prediction Field

Given the importance of the knowledge of SDPs for protein design, it is essential to use an economically feasible and accurate predictor. To this end, using machine learning (ML) methodologies in SDP prediction is very suitable, as ML tools would allow calculating dozens of SDP predictions in seconds. Though, the current ML-based approaches face many challenges. As an example, the majority of the sequence-based SDP prediction methods require a precalculated MSA file, together with subfamilies or subgroups definition. Here, caution should be taken as different MSA algorithms produce different alignment results based on varying parameters, which in the end will affect the final SDP list. Besides, dividing protein families into subfamilies requires expert knowledge. As another important limitation, the experimentally determined SDP datasets are rather small, which, in turn, prevents creating large-scale training of the feature-based methods. Construction of such large-scale SDP training datasets will make it possible to use deep learning algorithms, which have outperformed state-of-the-art methods in similar problems (LeCun et al., 2015; Zamora-Resendiz and Crivelli, 2019; Gao et al., 2020; Dai and Bailey-Kellogg, 2021). The energy-based methods, as presented in this work under the umbrella of HADDOCK refinement and MD simulations, could offer a refined SDP list, which can be tested experimentally. These approaches, however, take much longer time as they explicitly use structures and calculate forces acting on these structures. As an example, HADDOCK refinement of complexes can take up to half an hour, depending on the available computing resources. MD simulations, on the other hand, can take up to days or weeks, based on the dedicated number of computing cores used. Considering the pros and cons of both approaches, it is evident that new SDP prediction methods, which combine the advantages of both sequence- and structure-based methodologies, should be developed. However, until then, to predict SDPs, conservation-filtered HADDOCK refinement can be used in combination with structurally-filtered Multi-RELIEF predictions. Both of these approaches are easily accessible through web services. Their combination covers all of the conservation-filtered MD-based SDP predictions, without the requirement of heavy calculations.

## Method

### Sequence-Based Methods

**SDPpred** is an entropy-based SDP prediction method which utilizes mutual information to determine well-conserved residues within the same groups but differ between them (Kalinina et al., 2004). The equation to mutual information score for a column p in the alignment is given below:

$$I_{p}=\sum_{i=1}^{N}\sum_{a=1}^{20}f_{p}\left(\alpha ,i\right)\text{log}\left(\frac{f_{p}\left(a,i\right)}{f_{p}\left(a\right)f_{p}\left(i\right)}\right)$$

In this equation, *N* is the number of specificity groups, *a* is the amino acid type, *f*_*p*_(*i*) ratio of protein sequences belonging to group *i*. *f*_*p*_(*a*) is the number of occurrences of residue *a* in the whole alignment at position *p*. *f*_*p*_(*a*,*i*) is the number of occurrences of residue *a* in group *i* at position *p*. SDPpred calculates column-wise scores for each position in the MSA and outputs SDPs over the protein sequences. The server can be reached at http://monkey.belozersky.msu.ru/~psn/query.htm.

**Multi-RELIEF** a machine-learning based SDP prediction method which employs RELIEF algorithm to identify specificity determining residues (Kononenko, 2005; Ye et al., 2008). Multi-RELIEF algorithm requires predefined groups and their MSA as input. The aim of this method is to calculate a weight vector for each position in MSA. The weight vector is initialized with zeros at the beginning. At each iteration, a random sequence *seq* is selected and its nearest neighbors from the same class (i.e., hit(*seq*)) and opposite class (i.e., miss(*seq*)) are determined based on the Hamming distance. Subsequently, the weight of each residue is calculated with the following equation:

$$\text{w}\left[i\right]=w\left[i\right]-\frac{diff\left(seq\left[i\right],miss\left(seq\right)\left[i\right]\right)}{m}+\frac{diff\left(seq\left[i\right],hit\left(seq\right)\left[i\right]\right)}{m}$$

where

$$diff\left(a,b\right)=\left\{ \begin{array}{c} 0,a=b\\ 1,a\neq b \end{array}\right.$$

In the above equation, *i* represents the *i*th position in the weight vector or sequences and *m* represents the number of sequences. The algorithm outputs a weight vector whose length is the same as the number of positions in the alignment. Higher weights indicates the higher probability of being SDP for the corresponding position.

**Sequence Harmony** is another entropy-based SDP prediction method (Feenstra et al., 2007). It takes MSA and two user-specified groups as input and calculates relative entropy scores for each residue that shows degree of conservations. Sequence Harmony provides ranking of the entropy scores as outputs. Sequence Harmony and Multi-RELIEF methods are merged under the Multi-Harmony web server at https://www.ibi.vu.nl/programs/shmrwww/ (Brandt et al., 2010).

### Template-Based Modeling of Axl:ligand Complexes

LG1 domain of Pros1 was modeled with i-TASSER (Roy et al., 2010). Pros1-to-Gas6 structural alignment was carried out with FATCAT web-tool (Ye and Godzik, 2003) (by using the Gas6 coordinates of 2C5D). The final Axl:Pros1 coordinates were visualized and saved in PyMOL (Schrödinger, 2015). All Axl:ligand complexes were water refined with HADDOCK2.2 web server (van Zundert et al., 2016). The standard HADDOCK refinement protocol samples 20 models. These models slightly differ from each other as each one is refined with molecular dynamics simulation starting with a different initial velocity. In the end, the generated models are ranked with the HADDOCK score, which is a sum of electrostatics (E_Elec), van der Waals (E_vdW) and desolvation terms (E_desolv): 1.0. E_vdW+ 0.2. E_elec + 1.0. E_desolv. The top ranking four models, i.e., the best four models with the lowest HADDOCK scores, are offered as the final complex states. We generated 200 refined structures for each Axl:ligand complex. The top four ranking models were isolated as the final solutions.

HADDOCK refinement outputs residue-based energy scores of each complex (expressed in E_Elec, E_vdW and E_elec+E_vdW), deposited in *ene-residue.disp* file (can be found under HADDOCK output folder: structures/it1/water/analysis). This file describes the contributions of each interface amino acid to the intermolecular interaction. These residue-based HADDOCK energies were analyzed by using R (R Core Team, 2013) and Rstudio (RStudio Team, 2020).

### Molecular Dynamics Simulations

GROMACS 5.1.4 software and its tools were used to run molecular dynamics simulations (MD) and quality controls (e.g., temperature, pressure, RMSD, Rg analyses) (Van Der Spoel et al., 2005). The AMBER99SB-ILDN force field (Lindorff-Larsen et al., 2010) was used to parameterize the protein molecules, while the TIP3P water model was used to represent the solvent (Jorgensen et al., 1983). The simulation was run in a rhombic dodecahedron unit cell. The minimum periodic distance to the simulation box was set to be 1.4 nm. The mdp simulation files were adapted from https://github.com/haddocking/molmod-data (Rodrigues et al., 2016).

Before the production run, each complex was minimized in vacuum by using the steepest descent algorithm (Mandic, 2004). They were then solvated with the TIP3P water, together with neutralizing ions (51 NA+ and 48 CL- ions were added to neutralize Axl:Gas6, while 58 NA+ and 49 CL- ions were added to neutralize Axl:Pros1). The relevant topology files were edited according to the newly included NA+ and CL- ions. The second cycle of energy minimization was performed on the solvated systems. The solvent and hydrogen atoms were relaxed with a 20 ps long molecular dynamics simulation under constant volume where the temperature was equilibrated to 300 K (NVT). This was followed by 20 ps long molecular dynamics simulation under constant pressure where the pressure is equilibrated to 1 bar (NPT). As a last step before the production run, position restraints were released upon reduction of its force constant from 1,000 to 100, 100 to 10, and 10 to 0. To generate a parallel run of a given complex, random seed was changed before running the NVT step. The coordinates were written in every 10 ps. The integration time step was set to 2 fs.

For each Axl complex, we ran four independent (replica) MD simulations, totaling 1.6 microseconds. In each simulation, upon reaching 200 ns, the periodic boundary conditions were corrected. The system was stripped off solvent and ion atoms. The Root Mean Square Deviations (RMSDs) were calculated by using the average coordinates as a reference. After leaving the equilibration periods out (25 ns for Axl:Gas6 and 50 ns for Axl:Pros1), 350 snapshots for Axl:Gas6 and 300 snapshots from Axl:Pros1 were extracted.

### Interface Analysis

The interfacial hydrophobic contacts, hydrogen and salt bridges were calculated with *interfacea* python library (https://github.com/JoaoRodrigues/interfacea) (Rodrigues et al., 2019). *interfacea* classifies an inter-monomer interaction as hydrophobic, if there are at least two non-polar atoms within 4.4 Å. It considers a pairwise contact as a hydrogen bond, if a hydrogen donor (D) and acceptor (A) gets within 2.5 Å. It then filters D-H-A triplets with a minimum angle threshold (default 120 degrees). Finally, it classifies an interaction as a salt bridge if there are oppositely charged groups within 4.0 Å. For the interface classification as core and rim, EPPIC webserver was used (Duarte et al., 2012). Core residues are the ones that are buried at least in the protein structure (>95%). The rest of interface residues were counted as rim residues.

For comparing different simulations, the box-and-whisker statistics were generated with the standard boxplot function of R. The salt bridges were classified as stable if they were observed for >25% of a simulation time. They were classified as consistent if they were observed to be stable in all simulations.

## Data Availability Statement

The datasets presented in this study can be found in https://github.com/CSB-KaracaLab/Paralog_SDP.

## Author Contributions

All authors contributed to the conceptualization of this work, as well as to the writing of the manuscript. AR and TK performed the sequence-based analysis. TK performed simulations and all the structure-based analysis. JR devised and developed *interfacea* package. EK participated at all levels and supervised the work.

## Conflict of Interest

The authors declare that the research was conducted in the absence of any commercial or financial relationships that could be construed as a potential conflict of interest.
