# Supplementary material for: Predicting the Specificity- Determining Positions of Receptor Tyrosine Kinase Axl
Source: Front Mol Biosci. 2021 Jun 14;8:658906. doi: 10.3389/fmolb.2021.658906 (PMC8236827; doi:10.3389/fmolb.2021.658906)
Supplement: Supplementary file 1 [file Table_1.DOCX]

**SUPPLEMENTARY INFORMATION**

Predicting the Specificity-Determining Positions of Paralogous Complexes

**Tülay Karakulak^1,2,3,4,5^, Ahmet Sureyya Rifaioglu^6^, João P.G.L.M. Rodrigues^7^, Ezgi Karaca^1,2,*^**

^1^Izmir Biomedicine and Genome Center, 35330, Izmir, Turkey

^2^ Izmir International Biomedicine and Genome Institute, Dokuz Eylul University, 35340, Izmir, Turkey

^3^Institute of Molecular Life Sciences, University of Zurich, Zurich, Switzerland

^4^Department of Pathology and Molecular Pathology, University Hospital Zurich, Zurich, Switzerland

^5^Swiss Institute of Bioinformatics, Lausanne, Switzerland

^6^Department of Electrical - Electronics Engineering, İskenderun Technical University, 31200, Hatay, Turkey

^7^Department of Structural Biology, Stanford University School of Medicine, Stanford, California, United States of America

*** Correspondence:**Ezgi Karaca, ezgi.karaca@ibg.edu.tr

**SUPPLEMENTARY FIGURES**

**
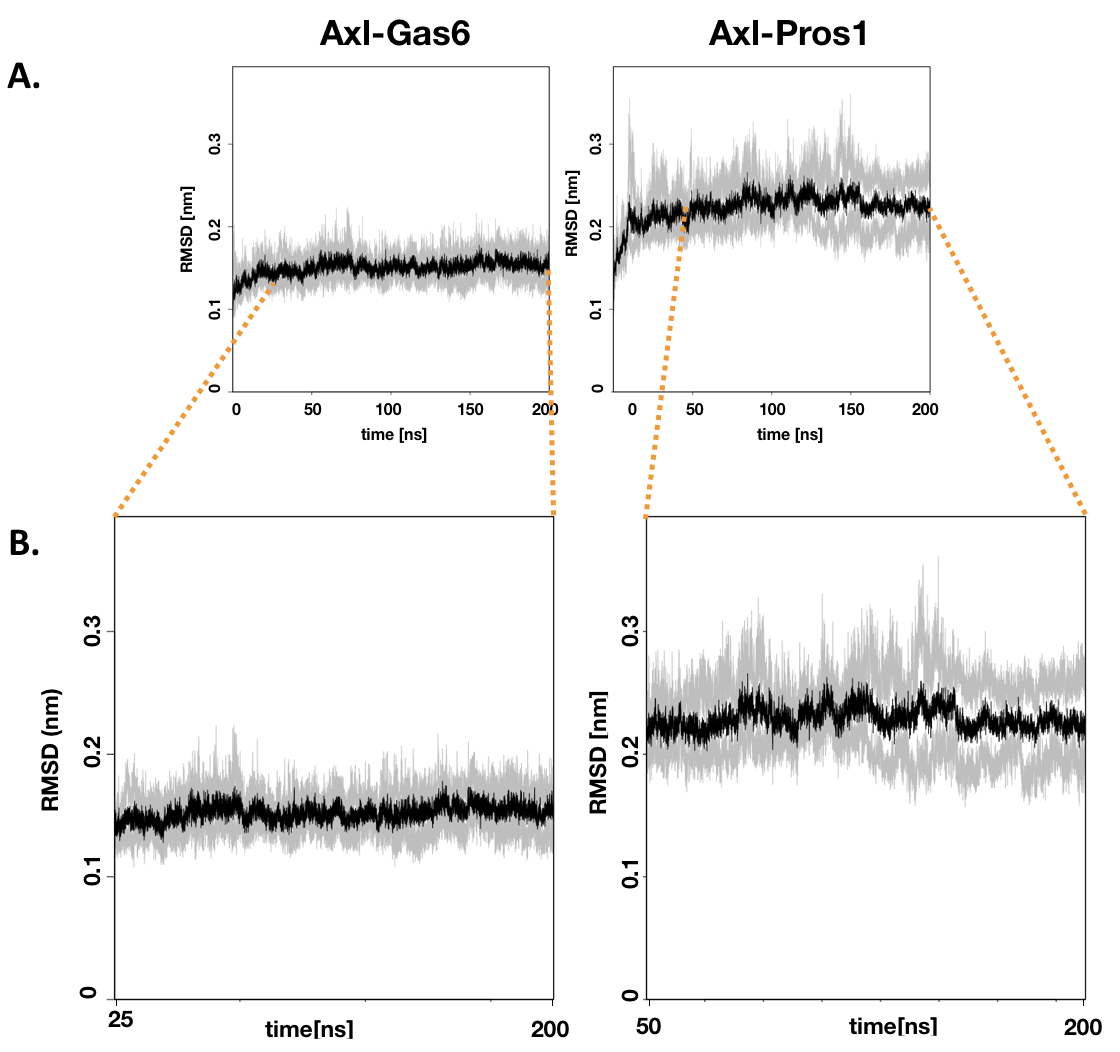
**

**Supplementary Figure 1.** Root mean square deviations (RMSDs) of Axl:Gas6 and Axl:Pros1 simulations from the average Axl:ligand structure. **(A)** Axl:Gas6 simulations reach equilibrium in 25 ns, whereas Axl:Pros1 simulations reach it in 50 ns. **(B)** During the production runs, Axl:Gas6 coordinates reflect 40% smaller mean RMSD (~0.15 nm) than Axl:Pros1 ones (~0.25 nm). Moreover, the RMSD values of Axl:Pros1 fluctuate between higher RMSD values (minimum: 0.15 nm and maximum: 0.36 nm for Axl:Pros1; minimum: 0.13 nm and maximum: 0.22 nm for Axl:Gas6).

**
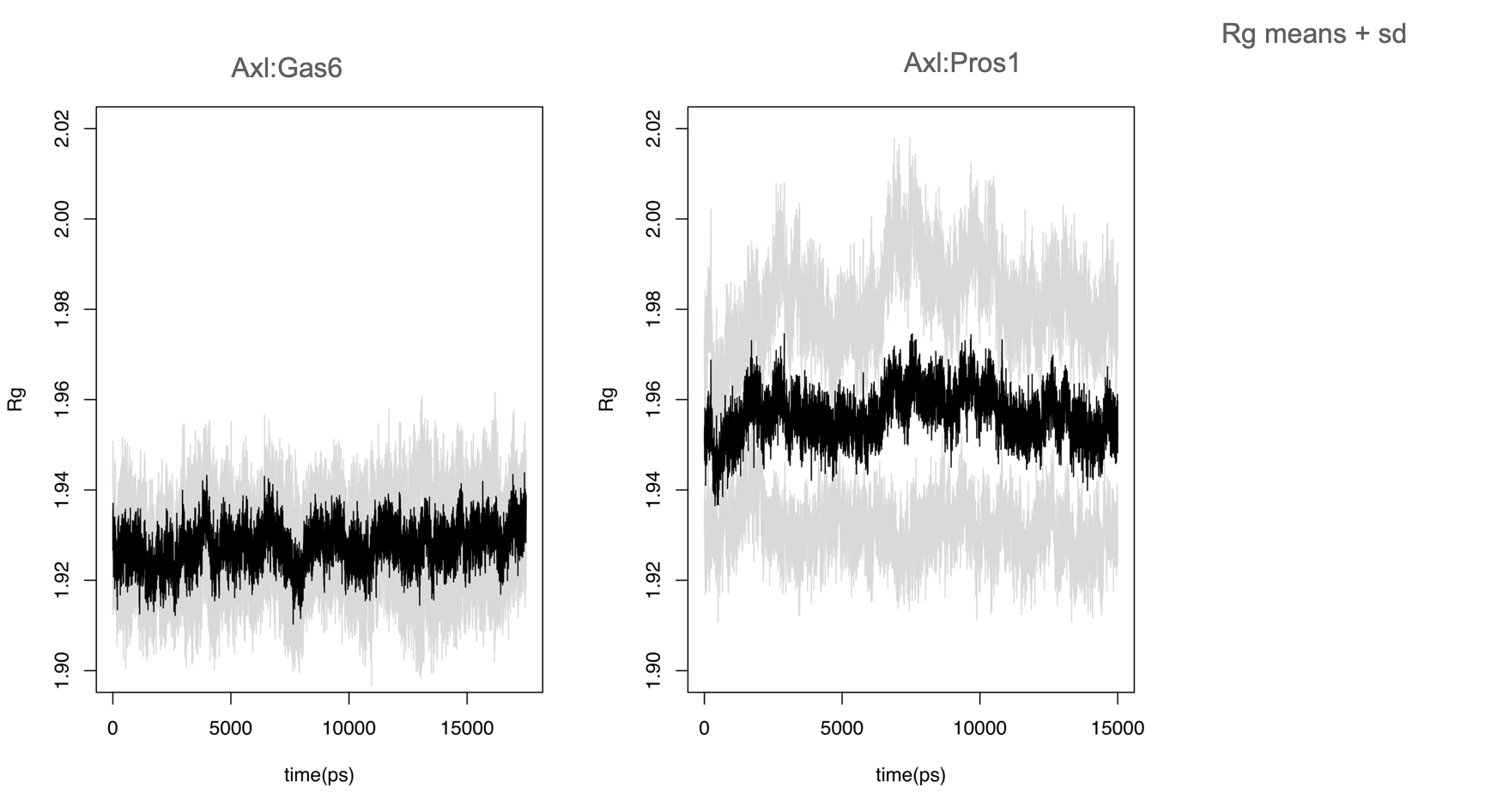
**

**Supplementary Figure 2.** The radius of gyration fluctuations (calculated over the backbone, expressed in nm) of Axl:Gas6 (left) and Axl:Pros1 (right) simulations.

**
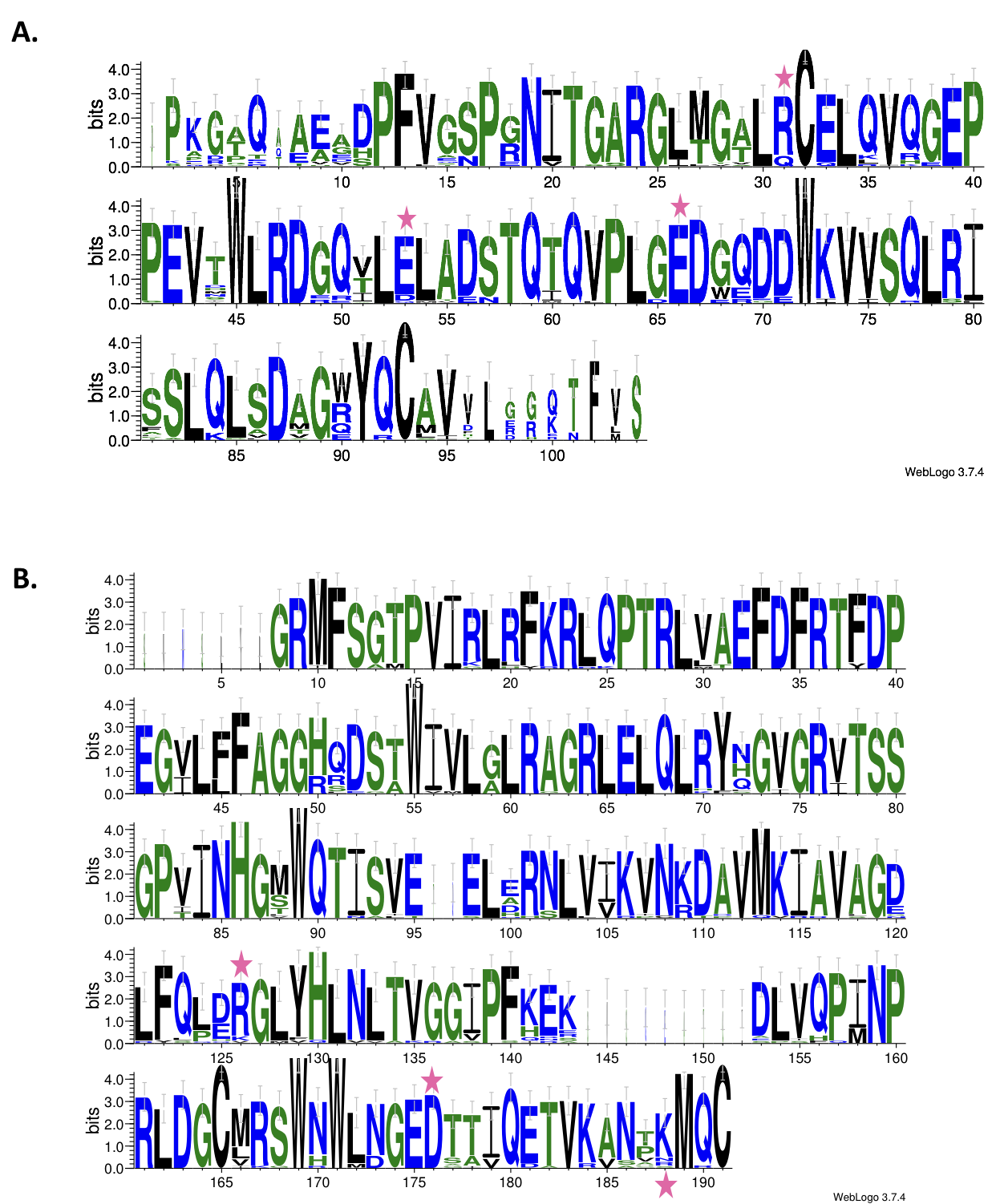
**

**Supplementary Figure 3.** Conservation of Axl SDPs and Gas6 residues across Axl and Gas6 orthologs.

**REFERENCES**

Hafizi, S., and Dahlbäck, B. (2006). Gas6 and protein S: Vitamin K-dependent ligands for the Axl receptor tyrosine kinase subfamily. FEBS J.

Shihab, H.A., Gough, J., Cooper, D.N., Day, I.N.M., and Gaunt, T.R. (2013a). Predicting the functional consequences of cancer-associated amino acid substitutions. Bioinformatics *29*, 1504–1510.

Shihab, H.A., Gough, J., Cooper, D.N., Stenson, P.D., Barker, G.L.A., Edwards, K.J., Day, I.N.M., and Gaunt, T.R. (2013b). Predicting the Functional, Molecular, and Phenotypic Consequences of Amino Acid Substitutions using Hidden Markov Models. Hum. Mutat. *34*, 57–65.

Tate, J.G., Bamford, S., Jubb, H.C., Sondka, Z., Beare, D.M., Bindal, N., Boutselakis, H., Cole, C.G., Creatore, C., Dawson, E., et al. (2019). COSMIC: The Catalogue Of Somatic Mutations In Cancer. Nucleic Acids Res. *47*, D941–D947.

Yanagihashi, Y., Segawa, K., Maeda, R., Nabeshima, Y., and Nagata, S. (2017). Mouse macrophages show different requirements for phosphatidylserine receptor Tim4 in efferocytosis.
